# Values Clarification Exercises to Prepare Nursing Students for Artificial Intelligence Integration

**DOI:** 10.3390/ijerph20146409

**Published:** 2023-07-20

**Authors:** Jennie C. De Gagne

**Affiliations:** School of Nursing, Duke University, Durham, NC 27710, USA; jennie.degagne@duke.edu

**Keywords:** artificial intelligence, nursing education, values clarification, ethical implications, patient-centered care, teaching strategy

## Abstract

Artificial intelligence (AI) is rapidly revolutionizing health care and education globally, including nursing practice and education. The responsible utilization of AI in a nursing context requires thoughtful consideration of its alignment with nursing values such as compassionate and patient-centered care provision, and respect for diverse perspectives. Values clarification, a vital teaching strategy in nursing education, can reinforce the foundational values and beliefs that guide nursing practice, thereby facilitating nurses’ critical evaluation of the ethical implications of AI implementation. The early introduction of values clarification into nursing education (a) provides students with a framework to prioritize and reflect on the impact of nursing values on their practice, (b) enables educators to make informed decisions and enhance teaching strategies, (c) contributes to the continual improvement of nursing education programs, and (d) fosters an ethical and values-driven approach to the integration of AI into nursing education and practice. This article examines the integration of values clarification into nursing education, offers strategies for nurse educators to integrate AI into their teaching toolkit effectively and ethically, and addresses concerns regarding potential misuses of AI.

## 1. Introduction

Artificial intelligence (AI) has garnered significant attention in the fields of education and health care as an innovation with the potential to revolutionize learning experiences and enhance patient outcomes [1]. In health care, AI applications such as predictive analytics, computer vision, and natural language processing provide real-time insights and can assist the analysis of vast medical datasets; these advancements play a vital role in reducing medication errors and improving patient monitoring [2]. In nursing education, AI has opened new possibilities, with virtual avatar apps and chatbots simulating interactive clinical scenarios to bolster students’ understanding of nursing concepts [3]. However, employing AI requires meticulous attention to responsible practices. 

Nurses leveraging new technologies, especially those infused with AI, need to be equipped to address the nuanced ethical implications tied to privacy and equity issues that may emerge from their use [1,4]. Consequently, nursing curricula must equip students with the skills needed to thrive in a technology-rich health care environment by integrating topics such as data literacy, cyberethics, and evaluation of the ethical implications of AI implementation. In this context, values clarification serves as a powerful teaching strategy that empowers students with a skillset that will of enduring value throughout their careers as they encounter new technologies and navigate their ongoing evolution [5]. This article aims to (a) discuss the effective use of values clarification as a strategy in nursing education and (b) provide nurse educators with strategies for effectively and ethically integrating AI into their teaching toolkit.

## 2. Values Clarification in Education

Values clarification is a well-established educational intervention that encompasses personal, sociocultural, and intercultural processes [6]. The process has been widely employed across various disciplines to enable individuals to recognize and apply their values effectively [5]. Values clarification enables students to cultivate greater self-awareness, enhance their decision-making skills, and develop a clearer sense of purpose and direction, thereby contributing to heightened satisfaction and fulfillment in their personal and professional endeavors [5].

Values clarification has gained popularity in education and other helping professions since the 1966 publication of “Values and Teaching” [7]. An early description of values clarification emphasized the skillful use of activities and questions to apply the valuing process to value-rich areas of individuals’ lives [7]. Its successful implementation can contribute to a deeper understanding of human growth and development, fostering better living and learning environments. Factors that commonly influence the values clarification process among students include personal experiences, family background, and cultural and societal norms as well as exposure to new ideas and perspectives [5]. Educators can play a pivotal role in supporting students on their values clarification journey by providing opportunities for self-reflection and exploration of personal values (e.g., courses in ethics, leadership, and role development; workshops and activities designed to foster critical thinking about individual beliefs and priorities).

The continued relevance of values clarification in the 21st century can be attributed to several factors: (1) in an increasingly complex world, students are required to develop critical thinking and sound decision-making skills, abilities that are significantly fostered by values clarification [6]; (2) in a world characterized by diverse and often conflicting values, students often strive to develop a sense of self-worth and confidence in their own beliefs, a process aided by values clarification; and (3) in a fast-paced world in which technology allows discourse and decision-making across global boundaries, students who understand their own values and those of others are better equipped to build strategic relationships and engage in effective communication [6]. Overcoming challenges such as professional jealousy, competition, and economic interests while promoting positive attitudes of collaboration and cooperation are essential goals of values clarification [7].

## 3. The Role of Values Clarification in Nursing Education

The need for values clarification in nursing education extends beyond geographical boundaries because nursing is a globally relevant profession that attracts students from diverse cultural backgrounds [8]. Nursing, as a profession, interacts with individuals from various cultural backgrounds, making it essential to empower students with the ability to identify and integrate their unique cultural values into their nursing practice. Values clarification plays a significant role in facilitating students in identifying and integrating their unique cultural values into their nursing practice. This process ultimately promotes culturally sensitive care and contributes to enhancing the overall quality of health care delivery.

Values clarification holds a crucial position in nursing education, serving as a vital component in the professional development of nursing students [9,10]. By emphasizing essential values such as patient-centered care, confidentiality, respect for patient autonomy, compassion, professionalism, evidence-based practice, interdisciplinary collaboration, empathy, and lifelong learning [9], values clarification equips students with guiding principles that are fundamental to delivering high-quality patient care and promoting positive health outcomes. In an era characterized by the increasing integration of AI in health care, the significance of values clarification becomes even more pronounced. It ensures that nursing students critically evaluate the ethical implications of AI and establish its alignment with patient-centered care and diverse perspectives.

The pedagogical design of nursing education is structured to equip students with the essential skills and knowledge required for safe and effective practice, while also aligning with the core values of the nursing profession [9]. Active participation in values clarification exercises offers significant benefits to students by fostering a deeper understanding of themselves as professionals [10]. Through these exercises, students are encouraged to articulate their emotions and values, enabling them to translate these values into action. Furthermore, meaningful interactions with patients and peers in clinical or simulated settings, as well as the analysis of narratives in educational environments, contribute to the gradual internalization of professional nursing values such as compassion, empathy, and caring. This personalized approach to self-reflection and heightened self-awareness enables students to firmly establish these core values as an integral part of their professional identity [10].

Nurse educators play a vital role in facilitating the development and internalization of professional values among nursing students through a combination of theoretical education and clinical experiences [9,10]. Clinical practice assumes particular significance as it allows students to observe and internalize the behaviors demonstrated by experienced nurses. Throughout their education, nursing students may encounter challenges in upholding professional values, including ethical dilemmas and conflicts with colleagues or supervisors [9]. Values clarification provides an indispensable tool for navigating these challenges.

## 4. Strategies for Values Clarification in AI Integration

Given the increasing integration of AI into health care, it is imperative to critically evaluate the ethical implications of AI and ensure that its utilization respects diverse perspectives and upholds principles of patient-centered care. The early introduction of values clarification provides students with a framework to prioritize their personal values and prepare for the ethical integration of AI into their practice. To support educators in this endeavor, various strategies that combine values clarification and AI integration can be employed effectively, fostering critical thinking, promoting ethical discussions, and preparing nursing students to navigate the complexities of AI use in their future practice.

### 4.1. Case-Based Learning Exercises

Case-based learning exercises offer a platform for students to explore the ethical considerations of AI use in nursing practice. For example, in a nursing ethics class, students can be presented with a case study involving the use of generative AI for task assignments [11]. Working in small groups, each of which is assigned a specific nursing value such as patient-centered care or equity, the student can discuss whether and how the use of AI aligns or conflicts with their assigned value with consideration given to the perspectives of patients, health care professionals, and the broader community.

During these discussions, students can analyze the ethical implications of AI utilization, assess the potential benefits and risks, and explore the impact on nursing practice and patient outcomes [12]. The aim is to foster critical thinking, reflection, and decision-making skills as students navigate the complexities of AI integration [13]. It is of utmost importance to establish and maintain an inclusive, respectful, and civil environment that encourages the sharing and respectful consideration of diverse perspectives throughout the process. Following these discussions, the entire class should reconvene to share their findings and compare perspectives. This collective reflection and exchange of ideas contribute to a deeper understanding of the ethical considerations associated with AI use in nursing practice. It also promotes collaborative learning and enables students to gain insights from their peers’ perspectives. By implementing case-based learning exercises, nursing educators can effectively engage students in analyzing real-world scenarios and encourage critical thinking regarding the ethical implications of AI integration in nursing practice [11,12,13].

### 4.2. Virtual Reality Simulation Exercises

Virtual Reality (VR) simulation strategies are rapidly emerging as a crucial component of students’ educational trajectory [14]. These strategies provide students with an enriched hands-on experience that is pivotal in their comprehensive understanding of the practical applications and limitations of AI in real-world settings [15]. The integration of VR simulations into values clarification exercises serves as a potent conduit for immersive learning. By sculpting activities that reflect the actual usage of AI in modern nursing practice, students are not merely observers but active participants.

Health care delivery is a collective endeavor, with collaboration at its core [15]. In this regard, VR simulations serve as an ideal platform for students to collaborate with other health care professionals in a virtual setting. This interactive experience hones their team building and communication skills, both of which are paramount for optimal health care outcomes [15,16]. For example, VR can immerse students in simulated disaster situations where the use of AI for patient triage and critical decision-making becomes necessary. Through these immersive exercises, students not only refine their practical skills but also cultivate the acumen required for making informed decisions under pressure [16]. Within this collaborative and highly simulated environment, students have the opportunity to probe the complexities of AI integration in the health care sector. They are encouraged to critically assess the interplay between AI and clinical decision-making, striving to strike a balance between technological aid and human intervention. This comprehensive preparatory approach furnishes students with the required competencies to adeptly navigate the intricate junction of AI and health care ethics.

### 4.3. Group Projects

Group projects provide nursing students with dynamic opportunities for values clarification through interactive learning experiences. In courses such as nursing informatics, where theoretical knowledge and practical skills are essential, group tasks can play a significant role. These tasks may involve designing AI-powered tools or systems for nursing practice, fostering creativity and teamwork [17]. Throughout the process, students engage in brainstorming to generate AI-powered solutions that uphold nursing values and ethics, deepening their understanding of the ethical considerations.

During their collaborative work, students actively participate in designing algorithms, analyzing patient data, and developing user-friendly interfaces. These tasks allow them to understand how AI can be applied responsibly and ethically in nursing practice. Such group projects foster a forum for discussions and debates on ethical considerations, encouraging students to develop AI solutions that are not only innovative but also ethically sound [18]. The true strength of these group projects lies in their ability to inspire values clarification amidst the pursuit of technical competence. They necessitate teamwork and effective communication skills that are tied intimately to nursing values such as collaboration, empathy, and respect [9,15]. As students grapple with the complexities of these group projects, they also enhance their AI literacy, a vital skill that will support ethical decision-making in their future careers [17].

### 4.4. Reflective Writing Exercises

Reflective writing exercises, facilitated through forums, provide an opportunity for students to critically reflect on their experiences with AI tools in nursing practice. The essence of reflective writing facilitates a deep level of cognitive and affective domains of learning, which is instrumental in supporting the internalization of values and ethical principles [19]. Instructors play a vital role by providing thought-provoking prompts that nudge students to scrutinize the ethical implications of AI usage. These prompts could focus on themes such as AI’s impact on patient autonomy, patient privacy, and the integration of AI with evidence-based practice. Such thoughtfully curated prompts can act as catalysts for deep reflection and values clarification.

The reflective process extends beyond the act of writing alone. Through feedback and guidance, instructors create a vibrant and responsive learning ecosystem [19]. This continual feedback loop aids students in discerning how their reflections can shape their clinical decision-making processes and navigate the ethical terrain associated with AI use efficiently. It bridges the gap between theoretical knowledge and practical applications, enabling students to construct connections between their academic understanding of AI and its real-world implementation within an ethical context. Ultimately, reflective writing exercises emerge as a powerful tool for values clarification, enabling students to deeply engage with the ethical underpinnings of AI integration in nursing practice, and subsequently nurturing a comprehensive, critical, and ethically informed perspective.

## 5. Potential Misuse and Limitations of Values Clarification

Despite its merit as an effective teaching strategy, values clarification is not immune to potential misuse and inherent limitations. One primary concern is the risk of reinforcing existing biases or promoting specific political or ideological agendas. In cases where instructors introduce personal agendas into the values clarification exercises, it may constrict students’ critical thinking skills, thereby restricting their ability to consider alternative perspectives. There is also the risk of misuse in the form of indoctrination [20]. By subtly encouraging students to adopt a predefined set of values without critical evaluation, instructors could lead to a lack of diversity in thought and a narrow-minded approach in nursing practice.

Approaching values clarification requires careful consideration, especially when attempting to address key differences in a single workshop session [6]. Without a comprehensive understanding of alternative options, divergent ideas, and different nuances or meanings assigned to the same values by various groups, the results may lead to superficial understanding, essentializing, or over-stereotyping. Although the linguistic translation of values is possible, accurately capturing their full complexity can be challenging [6].

Moreover, values clarification must be accompanied by evidence-based practice, which forms the foundation of quality patient care. While values are integral to nursing, they must be combined with evidence-based principles to ensure that patient care is grounded in sound scientific knowledge and best practices. When values clarification overly prioritizes personal beliefs over scientific evidence, there is a risk of compromising patient care. It is crucial, therefore, to use values clarification responsibly and ethically, aligning it with established evidence-based principles to promote patient-centered care. This requires a thoughtful balance between personal values and the objective evidence provided by research and practice guidelines.

It is important that nurse educators who are hesitant or lack confidence to use values clarification as a teaching strategy should proactively seek support and resources. These educators may understandably have concerns about initiating or facilitating open discussions on sensitive ethical topics. Engaging in professional development opportunities, attending workshops, and participating in training sessions can enhance educators’ confidence, competence, and comfort by facilitating values clarification exercises. In addition to investing professional development, the use of evidence-based resources and guidelines on incorporating values clarification into teaching can help to alleviate concerns about potential misuse. These resources provide educators with practical frameworks, strategies, and best practices for seamlessly integrating values clarification into the educational setting. Through this intentional implementation, educators can create a safe and conducive learning environment where students can freely explore and articulate their values, engage in meaningful discussions, and develop a deep understanding of the ethical considerations in nursing practice.

## 6. Conclusions

The integration of AI into nursing education and practice is a globally relevant topic that requires collaboration among health care professionals worldwide. While this article has provided insights into the use of values clarification as an essential tool in integrating AI into the curriculum for nurse educators, it is important to acknowledge its limitations. The lack of specific evaluations assessing the efficacy of values clarification exercises in the context of AI integration is attributed to limited studies in this area. Future research should focus on conducting rigorous evaluations to assess the impact and effectiveness of values clarification in AI integration in nursing education. Despite this limitation, this article serves as a starting point for nurse educators interested in incorporating values clarification into their teaching practices. By fostering an ethical and values-driven approach to the integration of AI in nursing, nurse educators can prepare future nurses to navigate the complexities, uphold ethical standards, and deliver high-quality patient care. Continued research and innovation in this field are essential to further our understanding and ensure responsible AI integration in nursing education.

## Data Availability

Not applicable.
